# Regulatory mechanism of Haa1p and Tye7p in *Saccharomyces cerevisiae* when fermenting mixed glucose and xylose with or without inhibitors

**DOI:** 10.1186/s12934-022-01822-4

**Published:** 2022-05-28

**Authors:** Bo Li, Li Wang, Jin-Yu Xie, Zi-Yuan Xia, Cai-Yun Xie, Yue-Qin Tang

**Affiliations:** 1 College of Architecture and Environment, Sichuan University, No. 24, South Section 1, First Ring Road, Chengdu, 610065 Sichuan China; 2 Institute of Applied Chemistry, Department of Chemical Engineering, Tsinghua University, Beijing, 100084 China; 3 Institute of New Energy and Low-carbon Technology, Sichuan University, No. 24 South Section 1 First Ring Road, Chengdu, 610065 Sichuan China; 4 Sichuan Environmental Protection Key Laboratory of Organic Wastes Valorization, No. 24 South Section 1 First Ring Road, Chengdu, 610065 Sichuan China; 5 Engineering Research Center of Alternative Energy Materials & Devices, Ministry of Education, No. 24 South Section 1 First Ring Road, Sichuan 610065 Chengdu, China

**Keywords:** *Saccharomyces cerevisiae*, *HAA1*, *TYE7*, Transcriptome, Inhibitor tolerance, Xylose fermentation

## Abstract

**Background:**

Various inhibitors coexist in the hydrolysate derived from lignocellulosic biomass. They inhibit the performance of *Saccharomyces cerevisiae* and further restrict the development of industrial bioethanol production. Transcription factors are regarded as targets for constructing robust *S. cerevisiae* by genetic engineering. The tolerance-related transcription factors have been successively reported, while their regulatory mechanisms are not clear. In this study, we revealed the regulation mechanisms of Haa1p and Tye7p that had outstanding contributions to the improvement of the fermentation performance and multiple inhibitor tolerance of *S. cerevisiae*.

**Results:**

Comparative transcriptomic analyses were applied to reveal the regulatory mechanisms of Haa1p and Tye7p under mixed sugar fermentation conditions with mixed inhibitors [acetic acid and furfural (AFur)] or without inhibitor (C) using the original strain s6 (S), the *HAA1*-overexpressing strain s6H3 (H), and the *TYE7*-overexpressing strain s6T3 (T). The expression of the pathways related to carbohydrate, amino acid, transcription, translation, cofactors, and vitamins metabolism was enhanced in the strains s6H3 and s6T3. Compared to C_H vs. C_S group, the unique DEGs in AFur_H vs. AFur_S group were further involved in oxidative phosphorylation, purine metabolism, vitamin B6 metabolism, and spliceosome under the regulation of Haa1p. A similar pattern appeared under the regulation of Tye7p, and the unique DEGs in AFur_T vs. AFur_S group were also involved in riboflavin metabolism and spliceosome. The most significant difference between the regulations of Haa1p and Tye7p was the intracellular energy supply. Haa1p preferred to enhance oxidative phosphorylation, while Tye7p tended to upregulate glycolysis/gluconeogenesis.

**Conclusions:**

Global gene expressions could be rewired with the overexpression of *HAA1* or *TYE7*. The positive perturbations of energy and amino acid metabolism were beneficial to the improvement of the fermentation performance of the strain. Furthermore, strengthening of key cofactor metabolism, and transcriptional and translational regulation were helpful in improving the strain tolerance. This work provides a novel and comprehensive understanding of the regulation mechanisms of Haa1p and Tye7p in *S. cerevisiae*.

**Supplementary Information:**

The online version contains supplementary material available at 10.1186/s12934-022-01822-4.

## Background

Bioethanol production using lignocellulosic biomass as feedstock could ease the pressure on fossil energy consumption and further advance carbon neutrality plans [1]. *Saccharomyces cerevisiae* has superior ethanol fermentation performance, but its xylose metabolism and stress tolerance are two key bottlenecks restricting the development of lignocellulosic bioethanol [2, 3]. The heterologous expression of xylose reductase and xylitol dehydrogenase (XR-XDH), or xylose isomerase (XI) endows *S. cerevisiae* with xylose metabolic ability, which improves the utilization of lignocellulose biomass [4]. However, xylose metabolism is severely inhibited in the presence of inhibitors generated during the pretreatment process [5–7]. Therefore, enhancing the robustness of xylose-metabolic *S. cerevisiae*, can not only improve the conversion efficiency of the fermentable sugars to ethanol but also reduce the stringent requirements in the biomass pretreatment process [8].

Various inhibitors (acetic acid, furfural, vanillin, etc.) are inevitably released in hydrolysate with the dissolution of the fermentable sugars in the pretreatment process [9, 10]. The robust strain could be constructed or screened by traditional domestication [11, 12], mutagenesis [13], transcriptome-guided genetic engineering [14, 15], etc. Although many studies have been devoted to improving the inhibitor tolerance of *S. cerevisiae*, ethanol production is still not ideal when using lignocellulosic biomass as feedstock due to the heterogeneous composition and the multifarious inhibitors in pretreated slurries [16, 17].

Transcription factors (TFs) can involve in the regulation of genes, which is a feasible target for constructing robust strains using genetic engineering. The ethanol productivity of *S. cerevisiae* overexpressing TF of *SFP1* or *ACE2* could be increased by 300–400% when fermenting in a synthetic medium with acetic acid and furfural [14]. Besides these two TFs, overexpressing *HAA1* [5, 18], *MSN2/4* [19], *TYE7* [5], *YAP1* [20, 21], etc. can also improve the inhibitor tolerance of *S. cerevisiae*.

Understanding the regulatory mechanism of TF is very important for improving the robustness of *S. cerevisiae*. At present, those revealed regulatory mechanisms of the key TFs are limited to the conditions with single sugar and single inhibitor [21–24]. While the endogenous regulation mechanisms of *S. cerevisiae* are different among various fermentable sugars (glucose, xylose, and mixed glucose and xylose) under the conditions with/without inhibitor [5, 14, 25–27]. Furthermore, *S. cerevisiae* is more sensitive to inhibitors in the xylose fermentation stage than that in the glucose fermentation stage when mixed sugars are fermented [6, 7]. Considering glucose, xylose, and inhibitors coexist in the pretreated lignocellulose slurry, it would be of practical guiding significance to reveal the regulatory mechanism of TFs in enhancing strain tolerance to mixed inhibitors when fermenting mixed sugar.

In our previous study, two tolerant strains s6H3 and s6T3 were constructed by respectively overexpressing *HAA1* and *TYE7* in the parental strain s6 using the CRISPR/Cas9 gene engineering method [5, 28]. Mixed glucose and xylose fermentation using a synthetic medium with/without inhibitor and using pretreated corn stover slurry showed that both Haa1p and Tye7p had outstanding contributions in improving the xylose fermentation and inhibitor tolerance performance (Additional file 1: Fig. S1 and S2). Haa1p, a transcriptional activator, is previously reported as an important TF involved in response to multiple stress factors [5, 18]. Haa1p regulates the transcription of a set of genes that mainly encode membrane proteins (*TPO2*, *TPO3*, *YRO2*, etc.) [29]. Some transcriptomic analyses have found that Haa1p is involved in the activation of acetic acid-responsive genes, such as those encoding protein kinases and multidrug resistance transporters, as well as those involved in lipid metabolism and nucleic acid metabolism [22, 23]. GO classification statistics revealed that *HAA1* is involved in cellular copper/iron ion homeostasis [30], and positive regulation of transcription by RNA polymerase II [23, 29, 31]. However, these reported regulatory mechanisms of Haa1p are mainly focused on weak acid inhibition, and glucose is used as the fermentation sugar. The intracellular regulation caused by Haa1p is unknown under mixed acetic acid and furfural stress during mixed glucose and xylose fermentation.

Tye7p, a transcriptional activator, contributes to glycolytic genes activation, such as *ENO1* and *ENO2* (enolase), *TDH* (glyceraldehyde-3-phosphate dehydrogenase), *PGK1* (phosphoglycerate kinase), *PGM1* (phosphoglycerate mutase), *PYK1* (pyruvate kinase), and *TPI1* (triosephosphate isomerase) [32, 33]. Researchers have revealed that Tye7p involves in the positive regulation of the glycolytic process and activates Ty1 mRNA transcription [34, 35]. However, the regulation mechanism of Tye7p in response to inhibitors has not been reported.

In the present study, the regulatory mechanisms of Haa1p and Tye7p under the conditions with/without the mixed acetic acid and furfural were studied by comparative transcriptomics analysis using the strains s6, s6H3, and s6T3 when fermenting mixed glucose and xylose [5, 28]. The core genes, the enriched KEGG pathways, and the key TFs involved in the performance improvement were screened out. Based on these important elements, the regulatory networks of Haa1p and Tye7p were plotted. These revealed regulation mechanisms could be helpful to understanding the positive perturbations of Haa1p and Tye7p on the transcriptome and provide targets for the rational design of gene circuits, which lays a foundation for the construction of more robust strain to fermenting xylose and tolerating mixed acetic acid and furfural. Furthermore, it provides biological information for adding tolerance-related TFs to the database of *S. cerevisiae*.

## Results

### Fermentation performance of strains s6H3 and s6T3

Compared with the parent strain s6, the strains s6H3 and s6T3 had much better fermentation performance in 10% YPDX (10 g/L yeast extract, 20 g/L peptones, 60 g/L glucose, and 40 g/L xylose, pH 5) medium with/without mixed acetic acid (2.4 g/L) and furfural (1.9 g/L) (Additional file 1: Fig. S1). The xylose consumption rate was 2.33 g/L/h (s6), 3.44 g/L/h (s6H3), and 2.96 g/L/h (s6T3) in the first 8 h fermentation without inhibitor, and 0.88 g/L/h (s6), 1.33 g/L/h (s6H3), and 1.29 g/L/h (s6T3) in the first 24 h fermentation with mixed acetic acid and furfural stress.

With the motivation to understand the regulation mechanism of transcription factors (TFs) Haa1p and Tye7p in enhancing the fermentation performance and inhibitor tolerance of the strains, we designed comparative transcriptomic experiments by focusing on mixed glucose and xylose fermentation with or without mixed acetic acid and furfural. The fermentation process with mixed sugars as carbon sources could be divided into the glucose fermentation stage and the xylose fermentation stage. The effect of glucose repression lasted after glucose depletion. The transcriptional response of the glucose stage caused disturbance to the subsequent xylose stage, resulting in specific transcriptomic profiles [27]. Considering the coexistence of glucose and xylose in the whole slurry of lignocellulosic biomass and much more sensitivity to inhibitors of the xylose fermentation compared to the glucose fermentation, the cells collected at 7 h were used for RNA extraction to best reveal the endogenous regulatory mechanisms of strains s6H3 and s6T3 during the xylose fermentation stage (Additional file 1: Fig. S1).

### Transcriptome profile of *S. cerevisiae *s6, s6H3, and s6T3

The identification names of strains s6 (S), s6H3 (H), and s6T3 (T) were C_S, C_H, and C_T under the condition without inhibitor (C), and AFur_S, AFur_H, and AFur_T under the condition with mixed acetic acid and furfural (AFur), respectively. The transcriptome data were aligned with *S. cerevisiae* S288C after quality control for the raw data (Additional file 1: Fig. S3, Table S1). The transcription levels of *ADY2*, *ATO2*, *BTN2*, *ENO1*, *ENO2*, and *HSP30* of all 18 RNA samples were analyzed by reverse transcription and quantitative real-time PCR (RT-qPCR), and the results were consistent with the results of the transcriptome analysis, suggesting that the transcriptomic results were reliable (Additional file 1: Fig. S4).

The fragments per kilobase of exon per million reads mapped (FPKM) values of *HAA1* and *TYE7* in strains s6, s6H3, and s6T3 under the conditions with/without mixed acetic acid and furfural were shown in Table 1. The FPKM values of *HAA1* and *TYE7* were significantly increased under the initiation of *UBI4* promoter (P_*UBI4*_), which has little fluctuation under mixed acetic acid and furfural stress. These results indicated that the expressions of *HAA1* and *TYE7* were upregulated by P_*UBI4*_. To reveal the regulation mechanisms of Haa1p and Tye7p, the differences in genome expression profiles of the groups C_H vs. C_S, AFur_H vs. AFur_S, C_T vs. C_S, and AFur_T vs. AFur_S were analyzed based on differentially expressed genes (DEGs) and Kyoto Encyclopedia of Genes and Genomes (KEGG) enrichment. The DEGs were filtered with a threshold of false discovery rate (FDR) < 0.05 and a fold change (Sample B/Sample A) ≥ 1.5. The KEGG enrichment analysis was accorded to the KEGG database, and the enriched pathways were filtered with a threshold of enrichment ratio (E) ≥ 0.1 and/or *P* < 0.05.

**Table 1:** FPKM value of each gene in the strains s6 (S), s6H3 (H), and s6T3 (T) under the conditions with (AFur) or without (C) mixed acetic acid and furfural stress

Gene	FPKM		
	C_S	C_H	C_T
*HAA1*	47.69 ± 1.70	616.92 ± 69.26	43.37 ± 3.49
*TYE7*	118.77 ± 2.51	194.41 ± 33.47	1035.36 ± 54.53
*UBI4*	738.21 ± 21.62	476.46 ± 34.02	683.29 ± 39.23

Gene	AFur_S	AFur_H	AFur_T
*HAA1*	93.81 ± 3.31	543.30 ± 18.32	83.28 ± 6.25
*TYE7*	207.31 ± 15.85	230.39 ± 12.09	1076.24 ± 11.18
*UBI4*	985.90 ± 20.96	890.65 ± 35.14	1057.87 ± 59.20

### C_H versus C_S and AFur_H versus AFur_S

**Fig. 1:**
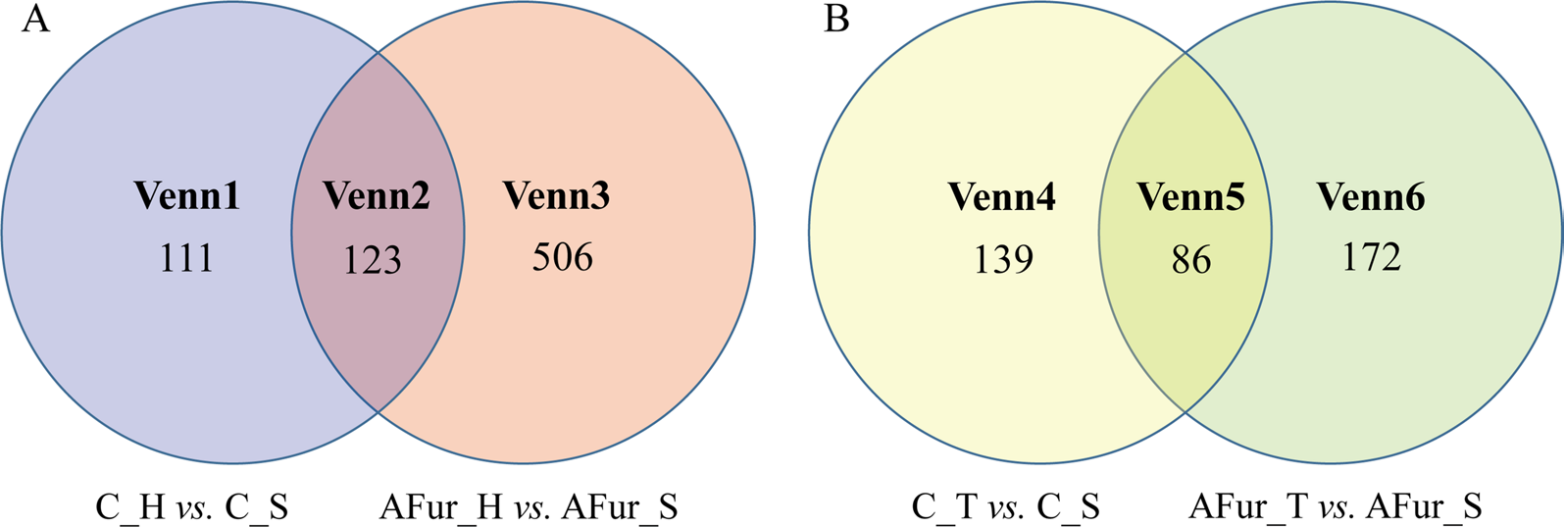
Venn diagrams of differentially expressed genes in C_H vs. C_S and AFur_H vs. AFur _S groups (**A**), and C_T vs. C_S and AFur _T vs. AFur _S groups (**B**)

The numbers of DEGs were 234 and 629 in C_H vs. C_S and AFur_H vs. AFur_S groups, respectively, in which 154 (234) and 517 (629) were upregulated. This result suggested that the transcription process was significantly affected by the overexpression of *HAA1*, especially in the presence of inhibitors. The distribution of the DEGs in these two groups were shown in Fig. 1A. The proportion of the shared DEGs (123) was 52.56% and 19.55% of the total DEGs in C_H vs. C_S and AFur_H vs. AFur_S groups, respectively.

**Fig. 2:**
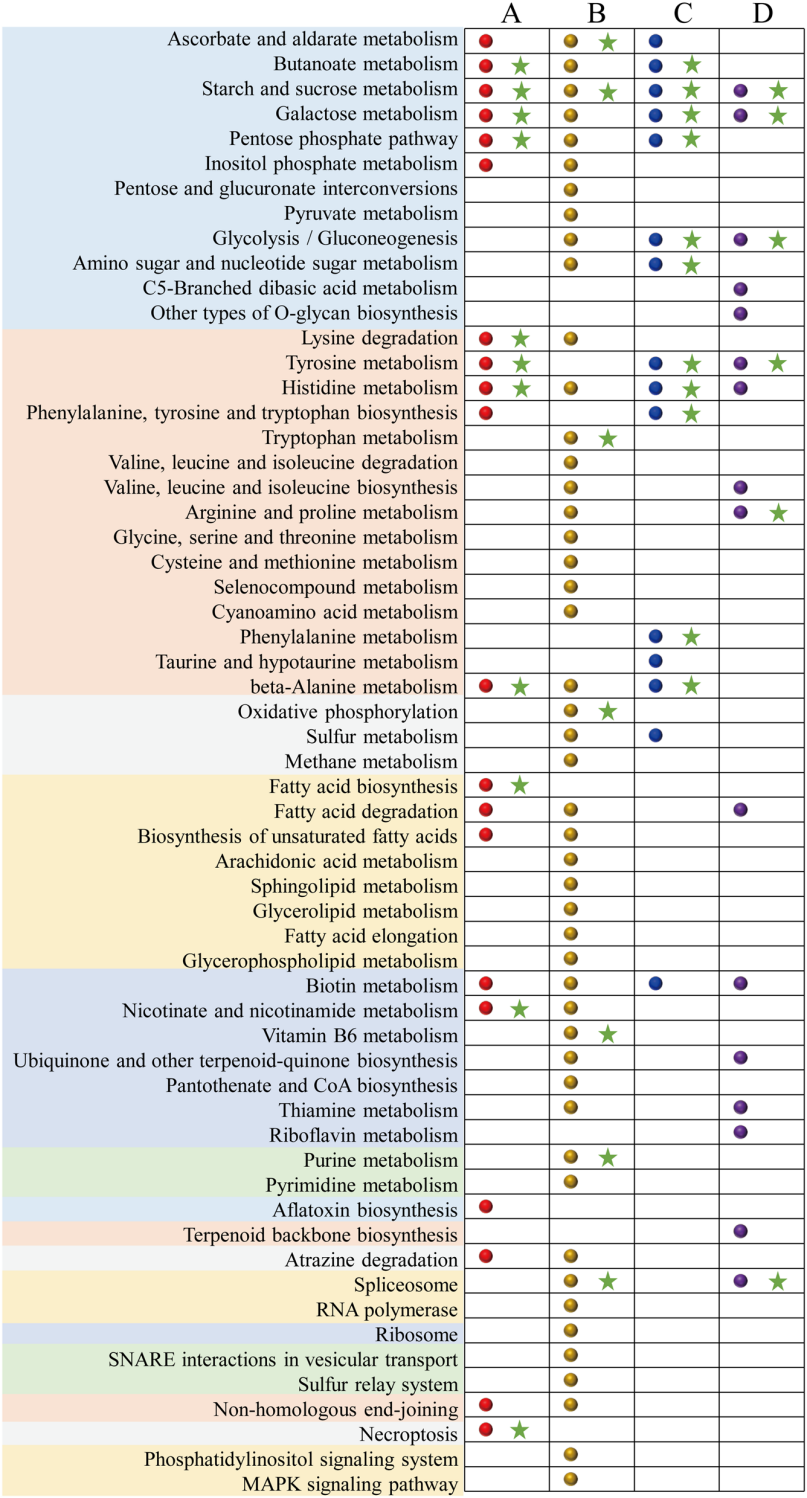
Bubble chart of the enriched KEGG pathways with a threshold of the enrichment ratio ≥ 0.1 in C_H vs. C_S (**A** red circle), AFur_H vs. AFur_S (**B** yellow circle), C_T vs. C_S (**C** blue circle), and AFur _T vs. AFur_S (**D** purple circle) groups. These circles followed by a green asterisk indicate that the pathway satisfies both enrichment ratio ≥ 0.1 and *P* < 0.05. The pathways on the left of the chart were classified by different background colors as carbohydrate metabolism, amino acid metabolism, energy metabolism, lipid metabolism, cofactors and vitamins metabolism, nucleotide metabolism, biosynthesis of other secondary metabolites, terpenoids and polyketides metabolism, xenobiotics biodegradation and metabolism, transcription, translation, folding, sorting and degradation, replication and repair, cell growth and death, and signal transduction

**Fig. 3:**
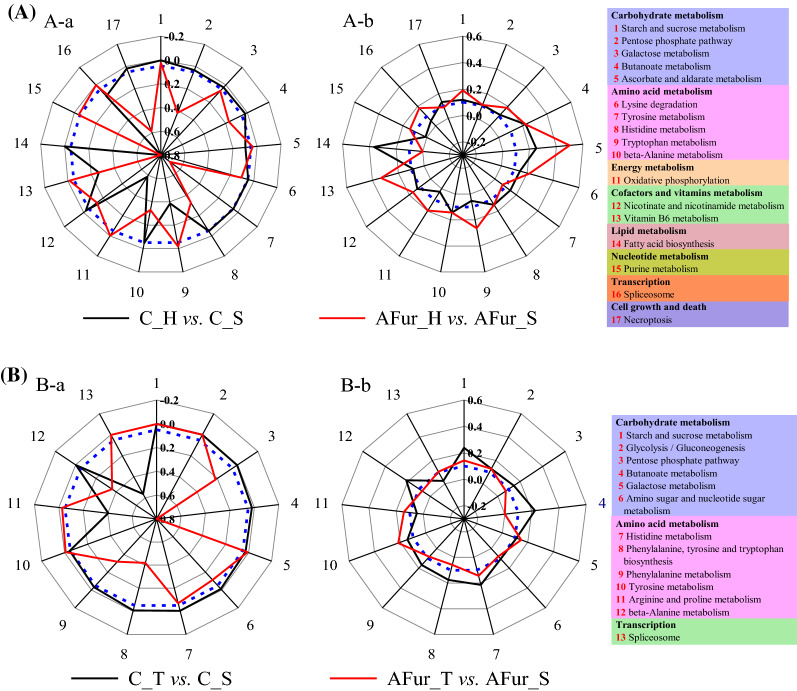
Radar map of the significantly enriched KEGG pathways. The pathways involved in C_H vs. C_S and AFur_H vs. AFur_S (**A**) groups. The pathways involved in C_T vs. C_S and AFur _T vs. AFur_S (**B**) groups. The radar map (**A-a** and **B-a**) was based on the *P* < 0.05 of each KEGG pathway, and the radar map (**A-b** and **B-b**) was based on the enrichment ratio ≥ 0.1 of each KEGG pathway. The values in the middle of the radar map **a** and **b** represent the *P* value [increased by 0.2 from the center (0.8) to the outside (− 0.2)] and the enrichment ratio [increased by 0.2 from the center (− 0.3) to the outside (0.6)] of each KEGG pathway, respectively. The blue circle with short dash in the radar map (**A** and **B**) represent *P* = 0.05 or enrichment ratio = 0.1

These DEGs were used for KEGG enrichment analysis. In AFur_H vs. AFur_S group, the number of the enriched pathways (E ≥ 0.1) was much larger than that in C_H vs. C_S group (Fig. 2). Screened the pathways filtered with *P* < 0.05 from those pathways that only meet E ≥ 0.1, two radar maps were drawn to reveal the regulation mechanism of Haa1p in improving the sugar consumption performance and inhibitor tolerance (Fig. 3A). 11 and 7 KEGG pathways were significantly enriched in C_H vs. C_S and AFur_H vs. AFur_S groups, respectively (E ≥ 0.1 and *P* < 0.05). There were 17 specific pathways when pathways from these two groups were combined into a whole. Only starch and sucrose metabolism was concurrently enriched in these two groups. In C_H vs. C_S group, the enriched pathways were mainly involved in carbohydrate metabolism (butanoate metabolism, galactose metabolism, and pentose phosphate pathway), amino acid metabolism (histidine metabolism, lysine degradation, tyrosine metabolism, and beta-alanine metabolism), lipid metabolism (fatty acid biosynthesis), cofactors and vitamins metabolism (nicotinate and nicotinamide metabolism), and cell growth and death (necroptosis). While carbohydrate metabolism (ascorbate and aldarate metabolism), amino acid metabolism (tryptophan metabolism), energy metabolism (oxidative phosphorylation), cofactors and vitamins metabolism (vitamin B6 metabolism), nucleotide metabolism (purine metabolism), and transcription (spliceosome) were significantly enriched in AFur_H vs. AFur_S group.

To explore new regulation perspectives of Haa1p in the conditions with or without inhibitors, the KEGG enrichment analysis of the DEGs in the different regions of the Venn plot was discussed in detail (Fig. 1; Table 2). The shared DEGs (123) of these two groups were mainly involved in carbohydrate and amino acid metabolism-related pathways. There were 111 DEGs (Venn1) that were unique in the group without inhibitors, which is less than half of the total DEGs (234). Fatty acid biosynthesis and aflatoxin biosynthesis were specifically enriched when these 111 unique DEGs were used for the analysis. Combined with the result of the KEGG enrichment analysis of the 234 DEGs, it indicated that fatty acid metabolism was of great significance in improving the xylose metabolism when fermenting without inhibitors. Under mixed acetic acid and furfural stress, the enriched pathways of the unique DEGs (506 DEGs, Venn3) were consistent with the result of all the 629 DEGs. These regional analyses emphasized the core position of oxidative phosphorylation, vitamin B6 metabolism, purine metabolism, and spliceosome in improving strain tolerance by Haa1p regulation.

**Table 2:** KEGG enrichment analysis for the six Venn regions of Fig. 1 (Enrichment ratio ≥ 0.1 and *P* < 0.05)

Type	Term	Enrichment ratio	*P* value
Venn1			
Lipid metabolism	Fatty acid biosynthesis	0.364	0.000
Biosynthesis of other secondary metabolites	Aflatoxin biosynthesis	0.500	0.027
Venn2			
Carbohydrate metabolism	Ascorbate and aldarate metabolism	0.250	0.047
Carbohydrate metabolism	Butanoate metabolism	0.222	0.005
Carbohydrate metabolism	Galactose metabolism	0.111	0.004
Carbohydrate metabolism	Inositol phosphate metabolism	0.105	0.021
Amino acid metabolism	Lysine degradation	0.143	0.012
Metabolism of cofactors and vitamins	Nicotinate and nicotinamide metabolism	0.125	0.003
Xenobiotics biodegradation and metabolism	Atrazine degradation	0.333	0.036
Venn3			
Amino acid metabolism	Tryptophan metabolism	0.211	0.043
Energy metabolism	Oxidative phosphorylation	0.169	0.003
Nucleotide metabolism	Purine metabolism	0.129	0.027
Metabolism of cofactors and vitamins	Vitamin B6 metabolism	0.250	0.049
Transcription	Spliceosome	0.169	0.003
Venn4			
Carbohydrate metabolism	Butanoate metabolism	0.222	0.009
Carbohydrate metabolism	Starch and sucrose metabolism	0.190	0.000
Energy metabolism	Sulfur metabolism	0.125	0.028
Amino acid metabolism	Histidine metabolism	0.143	0.022
Amino acid metabolism	Phenylalanine, tyrosine and tryptophan biosynthesis	0.118	0.032
Amino acid metabolism	Taurine and hypotaurine metabolism	0.333	0.049
Amino acid metabolism	beta-Alanine metabolism	0.214	0.001
Venn5			
Metabolism of cofactors and vitamins	Biotin metabolism	0.167	0.045
Venn6			
Glycan biosynthesis and metabolism	Other types of O-glycan biosynthesis	0.133	0.048
Amino acid metabolism	Arginine and proline metabolism	0.143	0.013
Amino acid metabolism	Tyrosine metabolism	0.143	0.042
Amino acid metabolism	Valine, leucine and isoleucine biosynthesis	0.143	0.042
Metabolism of cofactors and vitamins	Riboflavin metabolism	0.133	0.048

### C_T versus C_S and AFur_T versus AFur_S

The numbers of the DEGs were 225 and 258 in C_T vs. C_S and AFur_T vs. AFur_S groups, respectively, in which 136 (225) and 200 (258) were upregulated. Compared with the overexpression of *HAA1*, the overexpression of *TYE7* seemed to have a temperate influence on the transcription regulation in the presence of inhibitors. The proportion of the shared DEGs (86) was 38.22% and 33.33% of the total DEGs in C_T vs. C_S and AFur_T *v*s. AFur_S groups, respectively (Fig. 1B).

KEGG enrichment analysis showed that the number of the enriched pathways (E ≥ 0.1) in AFur_T vs. AFur_S group was larger than that in C_T vs. C_S group (Fig. 2). The pathways with the threshold of *P* < 0.05 were further screened to draw the radar map. As shown in Figs. 3B, 11 and 6 KEGG pathways were significantly enriched in C_T vs. C_S and AFur_T vs. AFur_S groups, respectively. There were 13 specific pathways when pathways from these two groups were combined into a whole. Starch and sucrose metabolism, glycolysis/gluconeogenesis, galactose metabolism, and tyrosine metabolism were commonly enriched in C_T vs. C_S and AFur_T vs. AFur_S groups. Besides these co-enriched pathways, butanoate metabolism, pentose phosphate pathway, and amino sugar and nucleotide sugar metabolism belong to carbohydrate metabolism, and histidine metabolism, phenylalanine, tyrosine and tryptophan biosynthesis, phenylalanine metabolism, and beta-alanine metabolism belong to amino acid metabolism, were specifically enriched in C_T vs. C_S group. The pathways, such as arginine and proline metabolism (amino acid metabolism) and spliceosome (transcription) were specifically enriched in AFur_T vs. AFur_S group.

The shared DEGs (86 DEGs, Venn5) were mainly involved in biotin metabolism, suggesting this pathway should occupy key loci in Tye7p regulation (Fig. 1; Table 2). For the unique DEGs (139 DEGs, Venn4) in C_T vs. C_S group, carbohydrate and amino acid metabolism-related pathways were still dominated in the list. In AFur_T vs. AFur_S group, in addition to carbohydrate and amino acid metabolism, riboflavin metabolism belonging to cofactors and vitamins metabolism was ranked in the front when the 172 unique DEGs (Venn6) were used for the enrichment analysis.

Combined with the results of the KEGG enrichment analysis for the total DEGs and the regional DEGs, the key role of central carbon metabolism and amino acid metabolism in improving strain performance was demonstrated (Figs. 2 and 3; Table 2). Besides these two categories, the pathways belonging to cofactors and vitamins metabolism, and transcription and translation processes also have a prominent contribution to enhancing the tolerance of the strain to mixed acetic acid and furfural.

### Differentially expressed transcription factors analysis

The DEGs regulated by Haa1p and Tye7p were mixed with some potential TFs. 13, 20, 8, and 5 potential TFs were picked up from the DEGs in C_H vs. C_S, AFur_H vs. AFur_S, C_T vs. C_S, and AFur_T vs. AFur_S groups, respectively (Additional file 1: Table S2). To reveal the regulation pattern of Haa1p and Tye7p, the genes regulated by Haa1p, Tye7p, and those possibly TFs were searched and summarized according to the YEASTRACT database. These searched genes have been experimentally demonstrated to be regulated by the TFs. Thus, we conducted a comparative analysis between these searched genes and the DEGs revealed in our research, considering the DEGs should be directly or indirectly regulated by Haa1p and Tye7p under corresponding conditions. It’s important to note that, some DEGs regulated by those potential TFs may undergo a cascade of regulation, that is the Haa1p and Tye7p regulated the expression of the potential TFs, and then these potential TFs regulated the expression of the target DEGs. Expect for this two steps regulation, these target DEGs may be directly regulated by Haa1p and Tye7p.

**Fig. 4:**
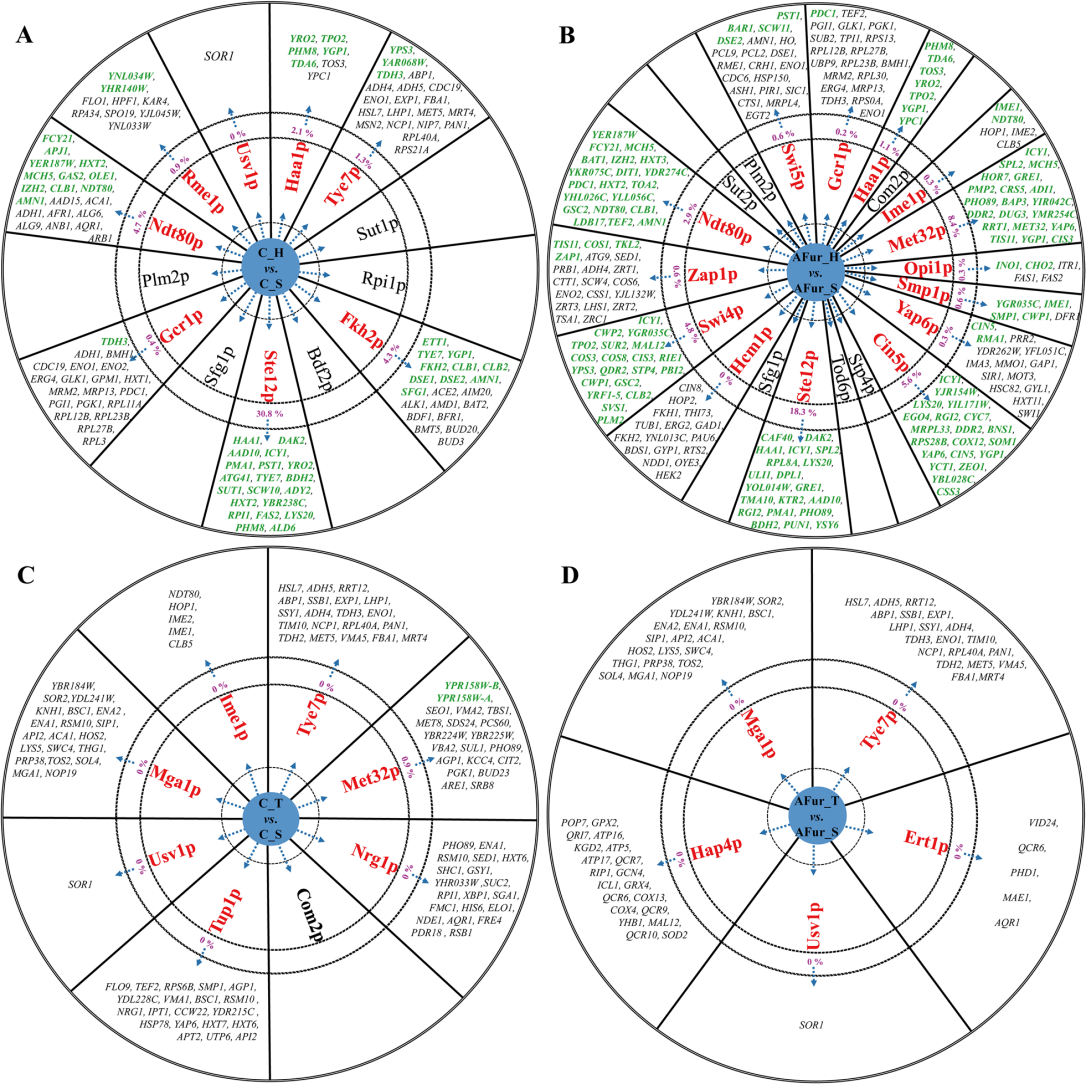
Schematic diagram of the genes regulated by the potential TFs in C_H vs. C_S (**A**), AFur_H vs. AFur _S (**B**), C_T vs. C_S (**C**), and AFur _T vs. AFur _S (**D**). The genes that are regulated by the TFs were searched and summarized according to the YEASTRACT database. The potential TFs in red indicate some genes are regulated by these TFs, and those in black indicate no gene is regulated by these TFs. The genes that are differentially expressed in each group are marked green. There are only 20 regulated genes listed in the diagram if the total number is greater than 20. The number represents the regulation ratio of each potential TFs, which is defined as the number of the DEGs regulated by the TFs to all the DEGs in each group

The genes regulated by the potential TFs were listed in Fig. 4 and Additional file 2: Table S3. Only 20 regulated genes were listed in the diagram if the total gene number is greater than 20. The regulation ratio of the potential TFs was defined as the number of the DEGs regulated by the TFs to all the DEGs in each group. The search results showed that some of the potential TFs (black font) do not have information on genes they regulate in the YEASTRACT database, possibly due to the regulatory mechanisms and functions of these TFs have not been reported to date.

### The regulation of Haa1p

There are only seven genes regulated by Haa1p via searching on the YEASTRACT database (Fig. 4A). Five of them were differentially expressed in C_H vs. C_S group. The regulation ratio of Haa1p was only 2.1% for the total DEGs (234), though it could be considered that the remaining 97.9% of DEGs are possibly directly or indirectly regulated by Haa1p. Except for the 5 TFs (Sut1p, Rpi1p, Bdf2p, Sfg1p, and Plm2p) that have no information on target genes in the database, the total regulation ratio of the other 7 TFs (Tye7p, Fkh2p, Ste12p, Gcr1p, Ndt80p, Rme1p, and Usv1p) was 35.47% for the total DEGs (234), in which the repeat DEGs were counted once. Among these 7 TFs, the regulation ratios of Ste12p, Ndt80p, and Fkh2p were 30.8% (72 DEGs), 4.7% (11 DEGs), and 4.3% (10 DEGs), respectively. Though it is possible that the DEGs regulated by these 6 TFs might be simultaneously regulated by Haa1p, to a large extent, Haa1p regulates the genomic expression by firstly regulating these TFs under the condition without inhibitors.

All the known genes regulated by Haa1p were DEGs in AFur_H vs. AFur_S group (Fig. 4B). Except for the 6 TFs (Com2p, Stp4p, Tod6p, Sfg1p, Sut2p, and Plm2p) that have no information on target genes in the database, the total regulation ratio of the other 13 TFs was 31.48% for the total DEGs (629), in which the repeat DEGs were counted once. Among these 13 TFs, the regulation ratio of Ste12p, Met32p, Cin5p, Swi4p, and Ndt80p was 18.3% (115 DEGs), 8.4% (53 DEGs), 5.6% (35 DEGs), 4.8% (30 DEGs), and 2.9% (18 DEGs), respectively. Obviously, under the condition with inhibitors, Haa1p affected the genome transcription by regulating much more TFs, which hence resulted in a much wider impact on genome transcription. Among these potential TFs, Ste12p, Sfg1p, Gcr1p, Plm2p, and Ndt80p regulated by Haa1p were the shared differentially expressed TFs under the conditions with and without inhibitors, while there were differences in the DEGs regulated by them in the two groups. These results indicated that the presence of inhibitors affected the regulation of Haa1p.

### The regulation of Tye7p

In the database, 27 genes are regulated by Tye7p (Fig. 4 C and D, Additional file 2: Table S3). While none of these 27 genes belonged to the DEGs in both groups with or without inhibitors. Two TFs (Usv1p and Mga1p) were the shared TFs in these two conditions. For all the target genes regulated by these potential TFs, only 2 genes in 336 genes regulated by Met32p were DEGs in C_T vs. C_S group. This indicated that the regulatory mechanisms of Tye7p and its regulated TFs during mixed sugar fermentation were possibly very different from those with the fermentation of glucose under the conditions of most researchers studied [34, 35]. Hence, further studies are needed to reveal the genes they regulate and the contributions of these TFs to xylose metabolism and inhibitor tolerance.

In conclusion, the number of genes actually regulated by these TFs, including Haa1p and Tye7p, may be much larger than those that now appeared in the YEASTRACT database. The information for the regulation targets of these potential TFs needs further investigation. While these potential TFs and the DEGs regulated by these potential TFs could be suggested as targets to further improve the xylose fermentation performance and the mixed acetic acid and furfural tolerance of *S. cerevisiae*.

## Discussion

**Fig. 5:**
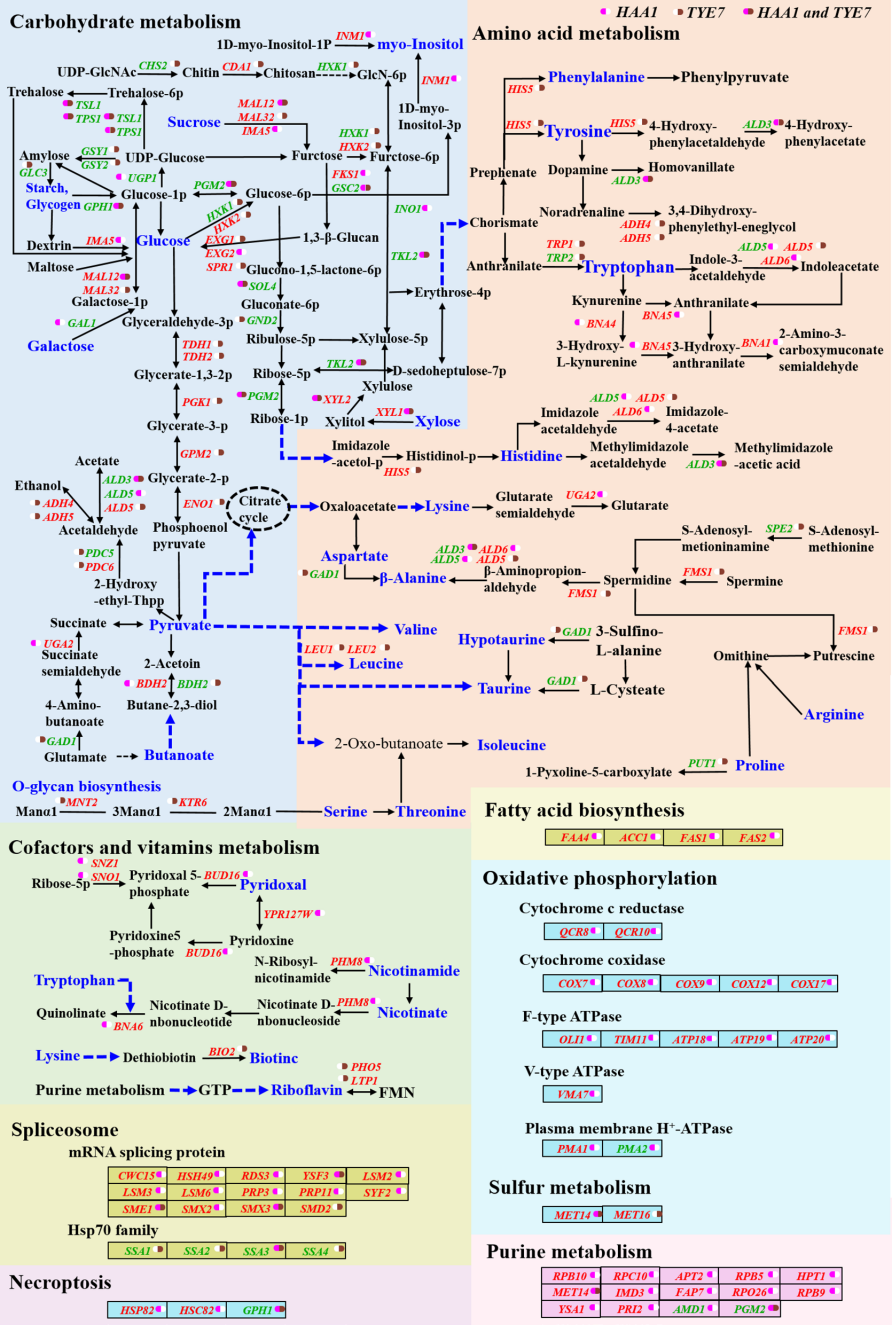
The regulatory mechanisms of Haa1p and Tye7p. The DEGs with red represent upregulated, and the DEGs with green represent downregulated. “” represents the DEGs were regulated by Haa1p, “” represents the DEGs were regulated by Tye7p, “” represents the DEGs were co-regulated by Haa1p and Tye7p

Even though only one TF (Haa1p or Tye7p) was overexpressed, positive perturbations were activated at the cellular level. The regulatory mechanisms of Haa1p and Tye7p were visually presented based on those key DEGs and KEGG pathways (Fig. 5, Additional file 1: Table S4). These diverse pathways and DEGs indicated the complex multi-level interactive response, which is the bottleneck that needs to be broken to construct robust strain. The key nodes involved in these complex networks could be used as primary targets to improve the strain performance by rationally designing genetic circuits.

Considering the consistency of the upregulation or downregulation of the DEGs, it was found that the regulation tendency of Haa1p or Tye7p was consistent, regardless of the inhibitors (Fig. 5). The pathways and genes involved in the regulation were diverse, in which the proportion of the pathways belonging to carbohydrate and amino acid metabolism was significantly higher than that belonging to other classifications. Carbohydrate-related genes are involved in energy metabolism. Oxidative phosphorylation is one of the key steps in ATP synthesis [36]. Tye7p preferred to regulate carbohydrate metabolism to obtain more energy, while Haa1p preferred to regulate oxidative phosphorylation to supplement more energy in addition to regulating carbohydrate metabolism.

Amino acids metabolism also occupied a large proportion in intracellular positive perturbations caused by the overexpressing of the two TFs (Fig. 5). Most of the DEGs involved in amino acid metabolism were up-regulated. Haa1p preferred to regulate lysine, tyrosine, histidine, tryptophan, and beta-alanine metabolism, while Tye7p preferred to regulate phenylalanine, tyrosine, tryptophan, histidine, arginine, proline, valine, leucine, isoleucine, beta-alanine metabolism, taurine, and hypotaurine metabolism. A previous study had demonstrated that the supplementation of arginine and lysine was helpful to increase acid tolerance in *Salmonella typhimurium* [37]. Dong et al. [38] had found that amino acids biosynthesis could be suppressed by acetic acid treatment. In these amino acid-related pathways, only those of tyrosine, histidine, tryptophan, and beta-alanine metabolism were the co-regulated pathways in the regulation of these two TFs. However, *ALD3* was the only co-regulated gene among all the DEGs involved in amino acid metabolism. These results indicated that Haa1p and Tye7p exhibited complex directionality in improving the strain performance.

For the cofactors and vitamins metabolism, the pathways of vitamin B6 metabolism, and nicotinate and nicotinamide metabolism were significantly enriched with the regulation of Haa1p, while biotin metabolism and riboflavin metabolism were significantly enriched with the regulation of Tye7p (Fig. 5). Nilsson et al. [39] found that the energy and cofactor (including both NADH and NADPH) utilization have a pivotal role in resisting mixed acetic acid and furfural stress. Vitamins have a positive effect on the growth and reproduction of *S. cerevisiae* [40]. The supplementation of vitamin E is beneficial for improving the fermentation and ethanol tolerance of *S. cerevisiae* [41]. The DEGs involved in these pathways were upregulated, suggesting the increasing flow of these pathways had indelible contributions to the performance enhancement.

Furthermore, spliceosomes belonging to transcription were also significantly enriched with the regulation of Haa1p and Tye7p (Fig. 5). Spliceosomes are crucial to the accurate transmission of genetic information [42]. The DEGs involved in pre-mRNA splicing were upregulated. The DEGs encoding chaperones of the Hsp70 family were downregulated. Besides these pathways, pathways of fatty acid biosynthesis, purine metabolism, and necroptosis were significantly enriched by the regulation of Haa1p. The upregulation of these pathways should play an important role in improving strain performance.

Haa1p has been confirmed to be involved in the response to acetic acid. About 80% of acetic acid-activated genes can be directly or indirectly regulated by Haa1p [22, 23]. The genes of *TPO2* and *TPO3,* and the membrane transporter genes have been demonstrated to be directly regulated by Haa1p [29, 43]. Mira et al. [22] revealed the regulatory mechanism of Haa1p in the presence or absence of acetic acid when glucose was used as the carbon source. They found that in a Haa1p-dependent manner, the genes encoded protein kinases and multidrug resistance transporters, and the genes involved in lipid metabolism and nucleic acid processing were activated in response to acetic acid. Compared with these results using glucose as the carbon source under acetic acid stress, the results of the present study revealed new regulatory targets of Haa1p in response to mixed acetic acid and furfural during the xylose fermentation stage when mixed glucose and xylose were fermented. Besides lipid metabolism, more genes involved in the metabolism of intracellular basic substances, such as carbohydrates, amino acids, nucleotides, energy, cofactors, and vitamins, were differentially expressed with the overexpression of Haa1p (Figs. 2 and 5).

The regulatory mechanism of Tye7p in response to typical inhibitors is rarely reported compared with Haa1p. Tye7p has been confirmed that it is mainly involved in the regulation of the glycolysis process [32, 33]. In the present study, the genes involved in glycolysis were also differentially expressed with the overexpression of *TYE7* in the presence or absence of acetic acid and furfural (Figs. 2 and 5). Besides glycolysis/gluconeogenesis, the pathways belonging to amino acids metabolism were significantly enriched.

The similar enriched pathways regulated by Haa1p and Tye7p are mainly distributed in carbohydrate, amino acid, energy, cofactors, and vitamins metabolism (Fig. 2, Additional file 1: Fig. S5, Table S4). Previous studies have demonstrated that the differential expression of these pathways has an indelible contribution to improving the fermentation and lignocellulose-derived inhibitors tolerance of *S. cerevisiae* [5, 14, 44, 45]. Furthermore, some pathways were specifically regulated by Haa1p or Tye7p. These specific pathways might have a similar contribution to performance enhancement, resulting in a similar improvement in the fermentation and inhibitor tolerance of strains s6H3 and s6T3 (Additional file 1: Fig. S1). This phenomenon is to some extent consistent with the fact that the performance improvement of strain s6H3T10 (*HAA1* and *TYE7* co-overexpressed) was “1 + 1 < 2” rather than “1 + 1 > 2” (Additional file 1: Fig. S2) [5].

Haa1p and Tye7p could regulate multiple genes and pathways, which is beneficial to the improvement of xylose fermentation and inhibitor tolerance. To further improve the performance of s6H3 and s6T3, those TFs regulated by Haa1p and Tye7p as shown in Fig. 4 should be potential targets, though their roles in performance improvement need firstly to be revealed. Generally, the improvement space is limited when regulating only one or several key genes or TFs [46]. Therefore, mining more target TFs is necessary for constructing more robust *S. cerevisiae* strains. It is critical to reveal more comprehensive and in-depth regulation mechanisms of TFs in *S. cerevisiae* under various fermentation conditions.

## Conclusions

Positive perturbations could be activated with the overexpression of *HAA1* and *TYE7* in strain s6, which promoted the xylose fermentation and inhibitor tolerance of the strain. Strengthening the energy, amino acid, cofactors and vitamins, and transcription and translation-related pathways were the key regulation point of Haa1p and Tye7p. The results not only contribute to the understanding of regulatory mechanisms of Haa1p and Tye7p, but also provide potential regulation targets possibly useful for further improving the performance of strain.

## Methods

### Strains

*Saccharomyces cerevisiae* strain s6, a xylose-fermenting flocculating industrial strain, was derived from KF7M-16 through adaptation using a xylose-containing medium in our previous study [28]. Strain KF7M-16 was constructed by genomic integration of two plasmids, pIUX1X2XK (contains *XYL1* (xylose reductase), *XYL2* (xylitol dehydrogenase) from *Scheffersomyces stipitis* as well as *XKS1* (xylulokinase) from *S. cerevisiae*) and pIWBGL1 (contains *BGL1* (β-glucosidase) from *Aspergillus aculeatus*) in the industrial flocculating yeast strain KF-7 [47]. Strains s6H3, s6T3, and s6H3T10 were obtained by overexpressing *HAA1* alone (*UBI4*_P_-*HAA1*-*HAA1*_T_), overexpressing *TYE7* alone (*UBI4*_P_-*TYE7*-*TYE7*_T_), and co-overexpressing *HAA1* and *TYE7* (*UBI4*_P_-*HAA1*-*HAA1*_T_, *UBI4*_P_-*TYE7*-*TYE7*_T_), respectively, in the parental strain s6 in our previous work [5].

### Media and cultural conditions

The strains s6, s6H3, and s6T3 were activated at 30 ℃ on the 2% YPD-agar plate (10 g/L yeast extract, 20 g/L peptones, 20 g/L glucose, and 15 g/L agar). After 24 h, a loopful of cells was transferred into a 500-mL conical flask with 100 mL of 5% YPD medium (10 g/L yeast extract, 20 g/L peptones, and 50 g/L glucose), and cultivated aerobically for 16 h (160 rpm, 30 °C) in a shaker. Fresh cells (0.5 g dry cell weight (DCW)) were collected by centrifugation (8000×*g*, 2 min), and transferred into 100 mL of 10% YPDX medium (10 g/L yeast extract, 20 g/L peptone, 60 g/L glucose, and 40 g/L xylose, pH 5) in a 300-mL conical flask. Flasks were incubated in a thermostat water bath (35 °C). The broth in flasks was stirred (200 rpm) using a magnetic stirring system. If necessary, acetic acid (2.4 g/L) and furfural (1.9 g/L) were added to the sterilized medium. The batch fermentation method was described previously [5].

### RNA extraction and sequencing

Cells used for RNA extraction were collected at 7 h from the control (without inhibitor, C), and mixed acetic acid (2.4 g/L) and furfural (1.9 g/L) (AFur) groups. The methods for extracting and measuring total RNA and RNA-seq were performed as previously described [5]. Three independent biological replicates were sequenced for each fermentation condition. The data were analyzed on the online platform Majorbio Cloud Platform (www.majorbio.com) after sequencing in Shanghai Majorbio Bio-pharm Technology Co., Ltd.

### Reverse transcription and quantitative real-time PCR (RT-qPCR)

To verify the accuracy of RNA-seq data, RNA samples used for transcriptome sequencing were also used for quantification of mRNA copies by RT-qPCR. Six genes, *ADY2*, *ATO2*, *BTN2*, *ENO1*, *ENO2*, and *HSP30*, with varied transcript abundance, were chosen to quantify the relative expression levels (Additional file 1: Table S5). The methods of reverse transcription (obtaining cDNA) and quantitative real-time PCR (qPCR) were performed as previously described [5]. The copy number of each gene was normalized using the *ACT1* expression level as a reference. The fold change was determined by the 2^−ΔΔCT^ method [48]. Each sample was run in triplicate, and each group was repeated three times. The value of RT-qPCR presented is the mean of the triplicate results.

### Transcriptome data analysis

Quantified gene expression results used FPKM (fragments per kilobase of exon per million reads mapped) as a unit. The gene filtered with a threshold of false discovery rate (FDR) < 0.05 and a fold change (Sample B/Sample A) ≥ 1.5 were considered as differentially expressed genes (DEGs). The KEGG (Kyoto Encyclopedia of Genes and Genomes) pathway terms with a *P* < 0.05 and enrichment ratio ≥ 0.1 were considered to be significantly enriched. The *P* value was calculated based on the hypergeometric distribution. The enrichment ratio of each KEGG pathway was the number of DEGs involved in each KEGG pathway to the number of total genes involved in each KEGG pathway. The potential transcription factors (TFs) were used to search for genes that have been experimentally shown to be regulated by the TFs from documented associations in the YEASTRACT database. The analysis was conducted as previously described [6].

### Analytical methods

The concentrations of glucose, xylose, and ethanol were determined as previously described [49]. Glucose and xylose were determined by HPLC equipped with a fluorescence detector (RF-10AXL). Ethanol was measured by GC with an FID detector and 2-propanol was used as the internal standard.

## Supplementary Information

**Additional file 1**: **Fig. S1.** The glucose (**A** and **D**), xylose** (B** and **E),** and ethanol** (C** and **F)** concentration curves of strains s6, s6H3, and s6T3 underthe condition without inhibitor (**A, B, **and** C**) and the condition with mixed acetic acid and furfural (**D, E, **and** F**). Black squaresrepresent strain s6; red circles represent strain s6H3; green upwards triangles represent strain s6T3 (Ref. 5). **Fig. S2.** Evaluation of inhibitor tolerance of strains by batch fermentation using 10% YPDX medium (**A**), 10% YPDX medium containing mixed acetic acid and furfural (2.4+1.9 g/L) (**B**),and pretreated corn stover slurry (Ref. 5) (**C**). Black squares represent strain s6; red circles represent strain s6H3; blue upwards triangles represent strain s6T3, green stars represent strain s6H3T10. **Fig. S3.** The cluster graph of expression pattern of DEGs in each group. C_S_1, C_S_2, and C_S_3 represent the three biological replicates of strain s6 in the control (C) group, Afur_S_1, Afur_S_2, and Afur_S_3 represent the three biological replicates of strain s6 in mixed acetic acid and furfural (Afur) group; C_H_1, C_H_2, and C_H_3 represent the three biological replicates of strain s6H3 in control (C) group, Afur_H_1, Afur_H_2, and Afur_H_3 represent the three biological replicates of strain s6H3 in mixed acetic acid and furfural (Afur) group; C_T_1, C_T_2, and C_T_3 represent the three biological replicates of strain s6T3 in control (C) group, Afur_T_1, Afur_T_2, and Afur_T_3 represent the three biological replicates of strain s6T3 in mixed acetic acid and furfural (Afur) group. **Fig. S4.** Validation of transcriptome data by RT-qPCR. The changed fold means the ratio of the expression level of a specific gene in the experimental group to that in the control group.The *ACT1* expression level was used as a reference in RT-qPCR. **Fig. S5.** Venn diagrams of the enriched pathways when overexpressed Haa1p and Tye7p, respectively. The black font represents the enriched pathways, and the red font represents the classification of each pathway. **Table S1.** The results of transcriptome data alignment with *S. cerevisiae* S288C. **Table S2.** The differently expressed TFs in C_H vs. C_S (234), C_T vs. C_S (225), AFur_H vs. AFur_S (629), and AFur_T vs. AFur_S (258) groups. **Table S4.** The differently expressed genes involved in the key KEGG pathway in C_H vs. C_S, AFur_H vs.AFur_S, C_T vs. C_S, and AFur_T vs. AFur_S groups. **Table S5.** The primers used for RT-qPCR.

**Additional file 2**: **Table S3.** The genes regulated by the potential TFs in C_H vs. C_S (13), C_T vs. C_S (20), AFur_H vs. AFur_S (8), and AFur_T vs. AFur_S (5) groups. The light yellow highlighted values are calculated by DEGs/genes (the number of the DEGs regulated by the TFs to the number of the genes regulated by the TFs) or DEGs/all the DEGs (the number of the DEGs regulated by the TFs to all the DEGs in each group). The green highlighted genes are the DEGs that regulated by the TFs.

## Data Availability

The raw sequencing data have been deposited in SRA under the accession number PRJNA785718. All data generated or analyzed during this study are included in this published article and its additional files.

## Abbreviations

KEGG: Kyoto encyclopedia of genes and genomes
FPKM: Fragments per kilobase of exon per million reads mapped
SGD: Saccharomyces genome database
FDR: False discovery rate
RT-qPCR: Reverse transcription and quantitative real-time PCR
DEGs: Differentially expressed genes
TFs: Transcription factors
E: Enrichment ratio
C: Fermentation condition without inhibitor
AFur: Fermentation condition with mixed inhibitors of acetic acid and furfural
s6H3: *HAA1*-overexpressing strain
s6T3: *TYE7*-overexpressing strain
