# Supplementary material for: Regulatory mechanism of Haa1p and Tye7p in Saccharomyces cerevisiae when fermenting mixed glucose and xylose with or without inhibitors
Source: Microb Cell Fact. 2022 May 28;21:105. doi: 10.1186/s12934-022-01822-4 (PMC9148474; doi:10.1186/s12934-022-01822-4)
Supplement: Supplementary file 1 — Additional file 1: Fig. S1. The glucose (A and D), xylose (B and E), and ethanol (C and F) concentration curves of strains s6, s6H3, and s6T3 underthe condition without inhibitor (A, B, and C) and the condition with mixed acetic acid and furfural (D, E, and F). Black squaresrepresent strain s6; red circles represent strain s6H3; green upwards triangles represent strain s6T3 (Ref. 5). Fig. S2. Evaluation of inhibitor tolerance of strains by batch fermentation using 10% YPDX medium (A), 10% YPDX medium containing mixed acetic acid and furfural (2.4+1.9 g/L) (B),and pretreated corn stover slurry (Ref. 5) (C). Black squares represent strain s6; red circles represent strain s6H3; blue upwards triangles represent strain s6T3, green stars represent strain s6H3T10. Fig. S3. The cluster graph of expression pattern of DEGs in each group. C_S_1, C_S_2, and C_S_3 represent the three biological replicates of strain s6 in the control (C) group, Afur_S_1, Afur_S_2, and Afur_S_3 represent the three biological replicates of strain s6 in mixed acetic acid and furfural (Afur) group; C_H_1, C_H_2, and C_H_3 represent the three biological replicates of strain s6H3 in control (C) group, Afur_H_1, Afur_H_2, and Afur_H_3 represent the three biological replicates of strain s6H3 in mixed acetic acid and furfural (Afur) group; C_T_1, C_T_2, and C_T_3 represent the three biological replicates of strain s6T3 in control (C) group, Afur_T_1, Afur_T_2, and Afur_T_3 represent the three biological replicates of strain s6T3 in mixed acetic acid and furfural (Afur) group. Fig. S4. Validation of transcriptome data by RT-qPCR. The changed fold means the ratio of the expression level of a specific gene in the experimental group to that in the control group.The ACT1 expression level was used as a reference in RT-qPCR. Fig. S5. Venn diagrams of the enriched pathways when overexpressed Haa1p and Tye7p, respectively. The black font represents the enriched pathways, and the red font represents the classificat [file 12934_2022_1822_MOESM1_ESM.docx]

**Title:**

Regulatory mechanism of Haa1p and Tye7p in *Saccharomyces cerevisiae* when fermenting mixed glucose and xylose with or without inhibitors

**Authors:**

Bo Li ^a,b^, Li Wang ^a^, Jin-Yu Xie^c^, Zi-Yuan Xia ^a, d^, Cai-Yun Xie ^a, d*^, Yue-Qin Tang ^a, c, d, e *^

**Affiliation:**

^a^ College of Architecture and Environment, Sichuan University, No. 24, South Section 1, First Ring Road, Chengdu, Sichuan 610065, China

^b^ Institute of Applied Chemistry, Department of Chemical Engineering, Tsinghua University, Beijing, 100084, China

^c^ Institute of New Energy and Low-carbon Technology, Sichuan University, No. 24 South Section 1 First Ring Road, Chengdu, Sichuan 610065, China

^d^ Sichuan Environmental Protection Key Laboratory of Organic Wastes Valorization, No. 24 South Section 1 First Ring Road, Chengdu, Sichuan 610065, China

^e^ Engineering Research Center of Alternative Energy Materials & Devices, Ministry of Education, China, No. 24 South Section 1 First Ring Road, Chengdu, Sichuan 610065, China

***Corresponding author**

Tel (fax): +86 2885990936; Email address: [xiecy@scu.edu.cn](mailto:xiecy@scu.edu.cn), [tangyq@scu.edu.cn](mailto:tangyq@scu.edu.cn)
















**Fig. S1** The glucose (**A** and **D**), xylose **(B** and **E),** and ethanol **(C** and **F)** concentration curves of strains s6, s6H3, and s6T3 under the condition without inhibitor (**A, B,** and **C**) and the condition with mixed acetic acid and furfural (**D, E,** and **F**). Black squares represent strain s6; red circles represent strain s6H3; green upwards triangles represent strain s6T3 (Ref. 5).










**Fig. S2.** Evaluation of inhibitor tolerance of strains by batch fermentation using 10% YPDX medium (**A**), 10% YPDX medium containing mixed acetic acid and furfural (2.4+1.9 g/L) (**B**), and pretreated corn stover slurry (Ref. 5) (**C**). Black squares represent strain s6; red circles represent strain s6H3; blue upwards triangles represent strain s6T3, green stars represent strain s6H3T10.


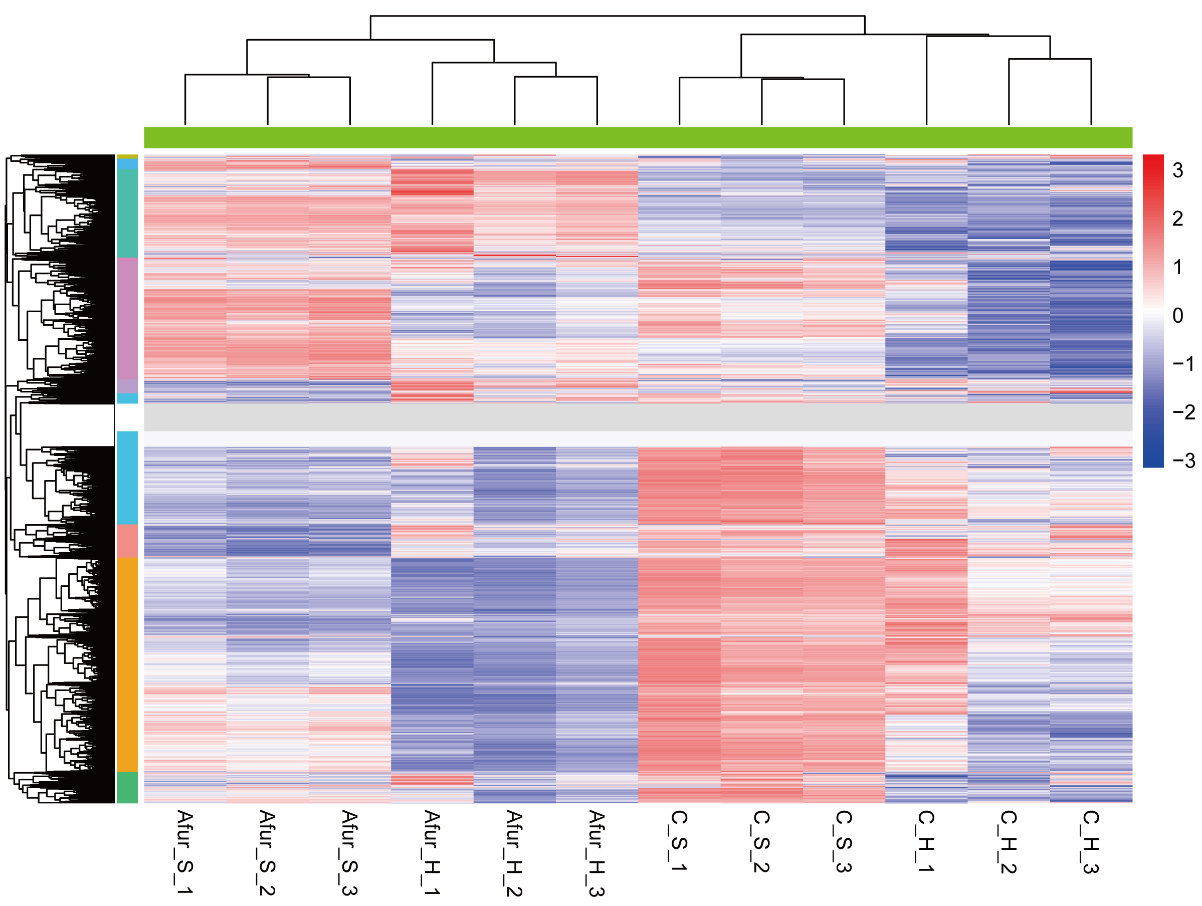


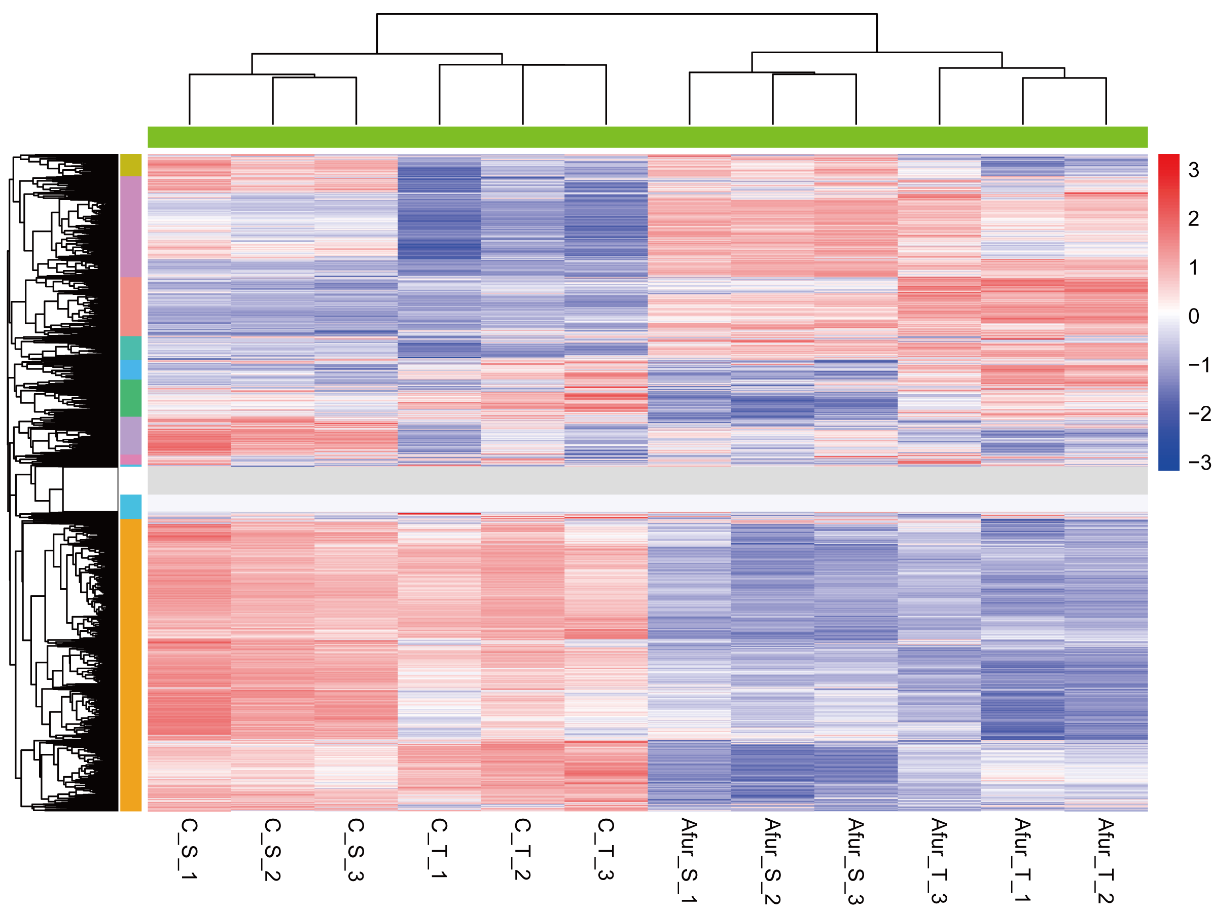


**Fig. S3.** The cluster graph of expression pattern of DEGs in each group. C_S_1, C_S_2, and C_S_3 represent the three biological replicates of strain s6 in the control (C) group, Afur_S_1, Afur_S_2, and Afur_S_3 represent the three biological replicates of strain s6 in mixed acetic acid and furfural (Afur) group; C_H_1, C_H_2, and C_H_3 represent the three biological replicates of strain s6H3 in control (C) group, Afur_H_1, Afur_H_2, and Afur_H_3 represent the three biological replicates of strain s6H3 in mixed acetic acid and furfural (Afur) group; C_T_1, C_T_2, and C_T_3 represent the three biological replicates of strain s6T3 in control (C) group, Afur_T_1, Afur_T_2, and Afur_T_3 represent the three biological replicates of strain s6T3 in mixed acetic acid and furfural (Afur) group.

**Fig. S4.** Validation of transcriptome data by RT-qPCR. The changed fold means the ratio of the expression level of a specific gene in the experimental group to that in the control group. The *ACT1* expression level was used as a reference in RT-qPCR.


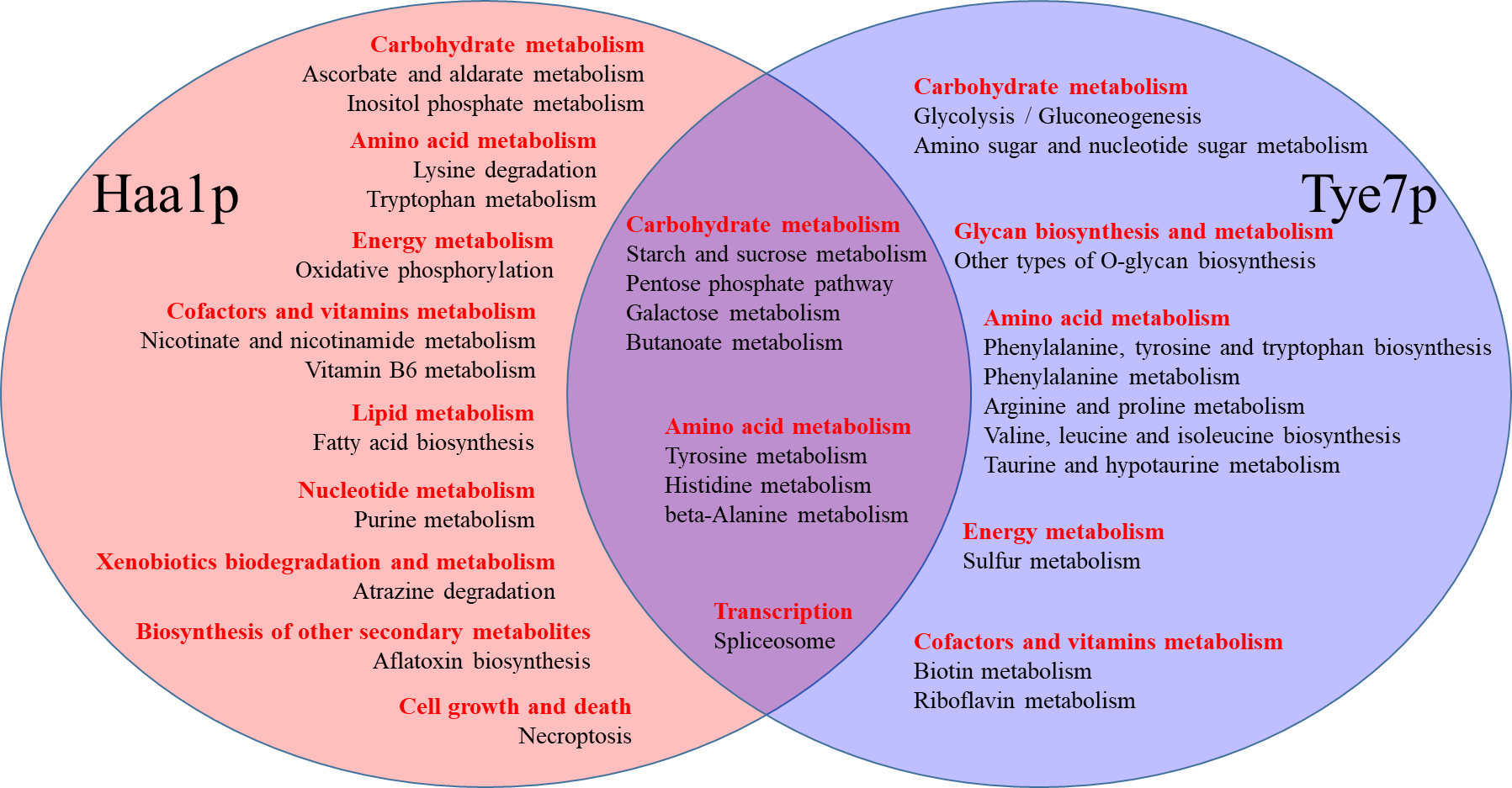


**Fig. S5.** Venn diagrams of the enriched pathways when overexpressed Haa1p and Tye7p, respectively. The black font represents the enriched pathways, and the red font represents the classification of each pathway.

**Table S1.** The results of transcriptome data alignment with *S. cerevisiae* S288C.

| Sample | Total reads | Total mapped | Multiple mapped | Uniquely mapped |
| --- | --- | --- | --- | --- |
| C_S_1 | 45065034 | 43108105(95.66%) | 5463312(12.12%) | 37644793(83.53%) |
| C_S_2 | 44841596 | 42945395(95.77%) | 6553494(14.61%) | 36391901(81.16%) |
| C_S_3 | 43095124 | 41152833(95.49%) | 6172529(14.32%) | 34980304(81.17%) |
| C_H_1 | 49102464 | 46935824(95.59%) | 9138316(18.61%) | 37797508(76.98%) |
| C_H_2 | 42759420 | 41045482(95.99%) | 10969725(25.65%) | 30075757(70.34%) |
| C_H_3 | 46416166 | 44572646(96.03%) | 12525135(26.98%) | 32047511(69.04%) |
| C_T_1 | 47893164 | 45817607(95.67%) | 9311001(19.44%) | 36506606(76.23%) |
| C_T_2 | 47683230 | 45562660(95.55%) | 7712886(16.18%) | 37849774(79.38%) |
| C_T_3 | 52111750 | 49837308(95.64%) | 9044514(17.36%) | 40792794(78.28%) |
| Afur_S_1 | 50859774 | 48241195(94.85%) | 5431380(10.68%) | 42809815(84.17%) |
| Afur_S_2 | 43082074 | 41001819(95.17%) | 5175467(12.01%) | 35826352(83.16%) |
| Afur_S_3 | 49156628 | 46736542(95.08%) | 5179811(10.54%) | 41556731(84.54%) |
| Afur_H_1 | 59402674 | 56394688(94.94%) | 8542093(14.38%) | 47852595(80.56%) |
| Afur_H_2 | 63707744 | 60606467(95.13%) | 11282509(17.71%) | 49323958(77.42%) |
| Afur_H_3 | 66188276 | 62742260(94.79%) | 8972655(13.56%) | 53769605(81.24%) |
| Afur_T_1 | 48593722 | 46103025(94.87%) | 6021278(12.39%) | 40081747(82.48%) |
| Afur_T_2 | 52206170 | 49410804(94.65%) | 5599981(10.73%) | 43810823(83.92%) |
| Afur_T_3 | 51069338 | 48292274(94.56%) | 4909833(9.61%) | 43382441(84.95%) |

**Table S2.** The differently expressed TFs in C_H *vs*. C_S (234), C_T *vs*. C_S (225), AFur_H *vs*. AFur_S (629), and AFur_T *vs*. AFur_S (258) groups.

| C_H *vs*. C_S | | | | | | |
| --- | --- | --- | --- | --- | --- | --- |
| Name | Description | FC | log_2_FC | Regulate | C_S_fpkm | C_H_fpkm |
| *HAA1* | Haa1p | 16.47 | 4.04 | up | 47.69 ± 1.70 | 616.92 ± 69.26 |
| *TYE7* | Tye7p | 2.09 | 1.06 | up | 118.77 ± 2.51 | 194.41 ± 33.47 |
| *SUT1* | Sut1p | 1.95 | 0.96 | up | 83.88 ± 3.98 | 130.21 ± 28.07 |
| *RPI1* | Rpi1p | 1.81 | 0.86 | up | 203.29 ± 17.98 | 237.65 ± 25.79 |
| *FKH2* | Fkh2p | 1.55 | 0.63 | up | 32.11 ± 4.42 | 39.18 ± 7.44 |
| *BDF2* | Bdf2p | 1.50 | 0.59 | up | 164.91 ± 4.17 | 192.35 ± 14.91 |
| *STE12* | Ste12p | 0.65 | -0.61 | down | 48.29 ± 0.70 | 24.93 ± 4.14 |
| *SFG1* | Sfg1p | 0.64 | -0.64 | down | 18.31 ± 2.49 | 9.27 ± 0.46 |
| *GCR1* | Gcr1p | 0.63 | -0.66 | down | 35.03 ± 1.19 | 17.30 ± 1.96 |
| *PLM2* | Plm2p | 0.63 | -0.67 | down | 110.04 ± 7.11 | 54.60 ± 7.78 |
| *NDT80* | Ndt80p | 0.58 | -0.78 | down | 17.20 ± 0.93 | 7.89 ± 1.02 |
| *RME1* | Rme1p | 0.51 | -0.97 | down | 11.43 ± 1.42 | 4.55 ± 1.03 |
| *USV1* | Usv1p | 0.50 | -1.00 | down | 58.56 ± 3.50 | 23.12 ± 2.59 |
| AFur_H *vs*. AFur_S | | | | | | |
| Name | Description | FC | log_2_FC | Regulate | AFur_S_fpkm | AFur_H_fpkm |
| *HAA1* | Haa1p | 6.47 | 2.69 | up | 93.81 ± 3.31 | 543.30 ± 18.32 |
| *COM2* | Com2p | 2.35 | 1.24 | up | 157.13 ± 2.57 | 329.87 ± 23.57 |
| *IME1* | Ime1p | 1.92 | 0.94 | up | 8.31 ± 0.40 | 14.18 ± 1.64 |
| *MET32* | Met32p | 1.80 | 0.85 | up | 158.79 ± 6.73 | 246.45 ± 32.84 |
| *OPI1* | Opi1p | 1.77 | 0.82 | up | 123.92 ± 2.78 | 194.73 ± 7.93 |
| *SMP1* | Smp1p | 1.74 | 0.80 | up | 53.66 ± 5.33 | 83.11 ± 2.61 |
| *YAP6* | Yap6p | 1.71 | 0.78 | up | 33.24 ± 0.15 | 50.56 ± 3.66 |
| *CIN5* | Cin5p | 1.70 | 0.76 | up | 161.15 ± 5.95 | 242.72 ± 13.14 |
| *STP4* | Stp4p | 1.56 | 0.65 | up | 466.23 ± 12.03 | 650.85 ± 35.46 |
| *TOD6* | Tod6p | 1.52 | 0.61 | up | 50.04 ± 2.07 | 68.13 ± 6.00 |
| *STE12* | Ste12p | 0.65 | -0.63 | down | 32.10 ± 1.07 | 18.59 ± 1.70 |
| *SFG1* | Sfg1p | 0.64 | -0.65 | down | 13.05 ± 1.38 | 7.35 ± 1.36 |
| *HCM1* | Hcm1p | 0.63 | -0.66 | down | 55.39 ± 5.02 | 31.27 ± 0.93 |
| *SWI4* | Swi4p | 0.62 | -0.69 | down | 21.67 ± 1.15 | 12.00 ± 0.65 |
| *ZAP1* | Zap1p | 0.61 | -0.71 | down | 41.92 ± 2.13 | 23.05 ± 1.34 |
| *NDT80* | Ndt80p | 0.59 | -0.75 | down | 13.90 ± 0.52 | 7.39 ± 0.06 |
| *SUT2* | Sut2p | 0.57 | -0.82 | down | 12.39 ± 0.49 | 6.19 ± 0.72 |
| *PLM2* | Plm2p | 0.57 | -0.82 | down | 58.02 ± 3.37 | 29.35 ± 2.16 |
| *SWI5* | Swi5p | 0.49 | -1.02 | down | 28.95 ± 2.61 | 12.76 ± 0.82 |
| *GCR1* | Gcr1p | 0.26 | -1.95 | down | 81.32 ± 2.63 | 23.09 ± 1.53 |
| C_T *vs*. C_S | | | | | | |
| Name | Description | FC | log_2_FC | Regulate | C_S_fpkm | C_T_fpkm |
| *TYE7* | Tye7p | 9.47 | 3.24 | up | 118.77 ± 2.51 | 1035.36 ± 54.53 |
| *MET32* | Met32p | 1.65 | 0.72 | up | 656.35 ± 12.00 | 1006.61 ± 97.70 |
| *NRG1* | Nrg1p | 1.62 | 0.70 | up | 276.27 ± 9.89 | 416.02 ± 7.03 |
| *COM2* | Com2p | 1.53 | 0.61 | up | 81.54 ± 2.93 | 115.33 ± 1.64 |
| *TUP1* | Tup1p | 0.65 | -0.61 | down | 377.67 ± 9.68 | 228.12 ± 20.59 |
| *USV1* | Usv1p | 0.54 | -0.90 | down | 58.56 ± 3.50 | 29.04 ± 3.43 |
| *MGA1* | Mga1p | 0.48 | -1.06 | down | 27.14 ± 3.15 | 12.04 ± 1.37 |
| *IME1* | Ime1p | 0.41 | -1.28 | down | 16.20 ± 1.04 | 6.22 ± 0.95 |
| AFur_T *vs*. AFur_S | | | | | | |
| Name | Description | FC | log_2_FC | Regulate | AFur_S_fpkm | AFur_T_fpkm |
| *TYE7* | Tye7p | 5.26 | 2.40 | up | 207.31 ± 15.85 | 1076.24 ± 11.18 |
| *ERT1* | Ert1p | 1.63 | 0.70 | up | 44.45 ± 3.30 | 73.46 ± 3.69 |
| *USV1* | Usv1p | 0.65 | -0.62 | down | 134.42 ± 13.90 | 88.66 ± 1.65 |
| *HAP4* | Hap4p | 0.65 | -0.62 | down | 332.39 ± 11.59 | 217.09 ± 7.20 |
| *MGA1* | Mga1p | 0.53 | -0.91 | down | 31.36 ± 2.45 | 16.88 ± 1.14 |

**Table S4.** The differently expressed genes involved in the key KEGG pathway in C_H *vs*. C_S, AFur_H *vs*. AFur_S, C_T *vs*. C_S, and AFur_T *vs*. AFur_S groups.

| KEGG pathway | DEGs |
| --- | --- |
| **C_H *vs*. C_S** | |
| Starch and sucrose metabolism | |
|  | ***EXG2***, ***IMA5***, ***FKS1***, *GPH1*, *PGM2* |
| Pentose phosphate pathway | |
|  | *TKL2*, *SOL4*, *PGM2* |
| Xylose metabolism | |
|  | ***XYL2*** |
| Galactose metabolism | |
|  | ***IMA5***, *GAL1*, *PGM2*, |
| Butanoate metabolism | |
|  | ***BDH2***, ***UGA2*** |
| Ascorbate and aldarate metabolism | |
|  | ***ALD6*** |
| Inositol phosphate metabolism | |
|  | ***INM1***, *INO1* |
| Lysine degradation | |
|  | ***ALD6*, *UGA2*** |
| Tyrosine metabolism | |
|  | ***UGA2***, *ALD3* |
| Histidine metabolism | |
|  | ***ALD6***, ALD3 |
| beta-Alanine metabolism | |
|  | ***ALD6***, *ALD3* |
| Nicotinate and nicotinamide metabolism | |
|  | ***PHM8***, ***UGA2***, ***BNA6*** |
| Fatty acid biosynthesis | |
|  | ***FAA4***, ***ACC1***, ***FAS2***, ***FAS1*** |
| Atrazine degradation | |
|  | ***DDI3*** |
| Aflatoxin biosynthesis | |
|  | ***ACC1*** |
| Necroptosis | |
|  | ***HSP82***, ***HSC82***, GPH1 |
| **AFur_H *vs*. AFur_S** | |
| Starch and sucrose metabolism | |
|  | ***EXG2***, ***IMA5***, ***MAL12***, *UGP1*, *TPS1*, *TSL1*, *GSC2*, *PGM2* |
| Xylose metabolism | |
|  | ***XYL1****,* ***XYL2*** |
| Ascorbate and aldarate metabolism | |
|  | ***ALD6***, *ALD5* |
| Butanoate metabolism | |
|  | ***BDH2,*** ***UGA2*** |
| Galactose metabolism | |
|  | ***IMA5***, *GAL1*, *PGM2* |
| Inositol phosphate metabolism | |
|  | ***INM1***, *INO1* |
| Lysine degradation | |
|  | ***UGA2***, ***ALD6*** |
| Tryptophan metabolism | |
|  | ***BNA1***, ***ALD6***, ***BNA5***, ***BNA4***, *ALD5* |
| Oxidative phosphorylation | |
|  | ***COX7***, ***ATP18***, ***PMA1***, ***OLI1***, ***COX17***, ***COX9***, ***COX12***, ***QCR8***, ***QCR10***, ***ATP19***, ***COX8***, ***VMA7***, ***TIM11***, ***ATP20***, *PMA2* |
| Vitamin B6 metabolism | |
|  | ***SNZ1***, ***BUD16***, ***SNO1***, ***YPR127W*** |
| Nicotinate and nicotinamide metabolism | |
|  | ***PHM8***, ***UGA2***, ***BNA6*** |
| Purine metabolism | |
|  | ***RPB10***, ***RPC10***, ***APT2***, ***RPB5***, ***HPT1***, ***MET14***, ***IMD3***, ***FAP7***, ***RPO26***, ***RPB9***, ***YSA1***, ***PRI2***, *AMD1*, *PGM2* |
| Spliceosome | |
|  | ***SME1***, ***YSF3***, ***RDS3***, ***LSM3***, ***LSM6***, ***HSH49***, ***LSM2***, ***SYF2***, ***SMX3***, ***SMX2***, ***PRP11***, ***PRP3***, ***CWC15***, *SSA3* |
| Atrazine degradation | |
|  | ***DDI3*** |
| **C_T *vs*. C_S** | |
| Starch and sucrose metabolism | |
|  | ***MAL32***, *TPS1*, *GSC2*, *HXK1*, *GPH1*, *GSY1*, *TSL1*, *GSY2*, *GLC3*, *PGM2* |
| Pentose phosphate pathway | |
|  | *GND2*, *SOL4*, *TKL2*, *PGM2* |
| Galactose metabolism | |
|  | ***MAL32***, *HXK1*, *PGM2* |
| Glycolysis / Gluconeogenesis | |
|  | ***TDH1***, ***ENO1***, ***ALD5***, ***PGK1***, ***TDH2***, *HXK1*, *ALD3*, *PGM2* |
| Xylose metabolism | |
|  | ***XYL1****,* ***XYL2*** |
| Amino sugar and nucleotide sugar metabolism | |
|  | ***CDA1***, *CHS2*, *HXK1*, *PGM2* |
| Butanoate metabolism | |
|  | *GAD1*, *BDH2* |
| Phenylalanine, tyrosine and tryptophan biosynthesis | |
|  | ***HIS5,*** ***TRP1***, *TRP2* |
| Tyrosine metabolism | |
|  | ***HIS5***, *ALD3* |
| Histidine metabolism | |
|  | ***ALD5***, ***HIS5***, ALD3 |
| Phenylalanine metabolism | |
|  | ***HIS5***, ALD3 |
| beta-Alanine metabolism | |
|  | ***ALD5***, *GAD1*, *ALD3* |
| Taurine and hypotaurine metabolism | |
|  | *GAD1* |
| Sulfur metabolism | |
|  | ***MET16***, ***MET14*** |
| Biotin metabolism | |
|  | ***BIO2*** |
| **AFur_T *vs*. AFur_S** | |
| Starch and sucrose metabolism | |
|  | ***EXG1***, ***MAL12***, ***MAL32***, ***HXK2***, ***SPR1***, *PGM2* |
| Galactose metabolism | |
|  | ***MAL12***, ***MAL32***, ***HXK2***, PGM2 |
| Glycolysis / Gluconeogenesis | |
|  | ***ADH4***, ***PDC6***, ***ADH5***, ***TDH2***, ***GPM2***, ***HXK2***, *PDC5*, *PGM2* |
| Xylose metabolism | |
|  | ***XYL1*** |
| Other types of O-glycan biosynthesis | |
|  | ***MNT2***, ***KTR6*** |
| Arginine and proline metabolism | |
|  | ***FMS1***, *SPE2*, *PUT1* |
| Tyrosine metabolism | |
|  | ***ADH4***, ***ADH5***, ***HIS5*** |
| Valine, leucine and isoleucine biosynthesis | |
|  | ***LEU2***, ***LEU1*** |
| Spliceosome | |
|  | ***YSF3***, ***SMX3***, ***SME1***, ***SMD2***, *SSA1*, *SSA2*, *SSA3, SSA4* |
| Riboflavin metabolism | |
|  | ***PHO5***, ***LTP1*** |
| Biotin metabolism | |
|  | ***BIO2*** |

**Table S5.** The primers used for RT-qPCR.

| Target gene | Primer | Sequence (5’~3’) |
| --- | --- | --- |
| *ACT1* | Rt-ACT1 F | ATGCAAACCGCTGCTCAA |
|  | Rt-ACT1 R | AGTTTGGTCAATACCGGCAGA |
| *ADY2* | Rt-ADY2 F | CCTTCGCGTTGACGACATT |
|  | Rt-ADY2 R | ACCAAACCACCATAAAACATAGCAC |
| *ATO2* | Rt-ATO2 F | CGCAAATCCTGCTCCACTA |
|  | Rt-ATO2 R | GCACACCCAACAACAACATT |
| *BTN2* | Rt-BTN2 F | CGGAGAAAGCGAAAGAACCA |
|  | Rt-BTN2 R | TGGCAGCTTTTTCCTGTTCTG |
| *ENO1* | Rt-ENO1 F | GCTTTCGTTAAGGCTAACATTG |
|  | Rt-ENO1 R | AAGAAACACCCAAGATAGCG |
| *ENO2* | Rt-ENO2 F | CGCTATCTTGGGTGTCTCCA |
|  | Rt-ENO2 R | GCACCAGTTGGAGCAATCAT |
| *HSP30* | Rt-HSP30 F | TTCAACCAGACGGTGAGGCTA |
|  | Rt-HSP30 R | CCTTGGCAATTTGCCATCA |
